# Adult Attachment Dimensions: Differential Effects on Physiological and Subjective Response During the Recollection of Childhood Memories

**DOI:** 10.5964/ejop.v16i4.1892

**Published:** 2020-11-27

**Authors:** Pierpaolo Congia

**Affiliations:** a Cognitive Psychotherapy Training School (ATC), Cagliari, Italy; Department of Psychology and Counselling, Webster University Geneva, Geneva, Switzerland

**Keywords:** attachment, parent–child relations, interview, psychophysiology, stress and coping

## Abstract

The aim of this study was to explore the relationship between adult attachment dimensions and emotional response induced by the recall of potentially painful memories from childhood. A convenience sample of 100 women responded to an interview that focused on experiences with their caregivers during childhood, and a control interview, in counterbalanced order. Skin conductance level (SCL), heart rate (HR), heart rate variability (HRV), as well as subjective distress measures were collected. Results from generalized linear mixed model indicated that individuals high in avoidance showed a pattern of SCL increase from baseline that persisted during rest phases regardless of the topic addressed. Attachment dimensions did not affect HR, neither alone nor interacting with the interviews content, whereas baseline resting vagal tone was the most important factor. No attachment dimensions effects were observed on subjective measures of emotion; the time-varying vagal tone during rest phases did not moderate their relationships. Limited evidence was observed in support of the hypothesis that attachment Avoidance and Anxiety are associated with distinct physiological regulation profiles during the recall of potentially painful childhood memories.

The recollection and elaboration of memories regarding painful episodes occurred during childhood are critical components of the psychotherapeutic process. What personality characteristics determine subjective and physiological responses and how do they interact with the content of the psychotherapy session? The attachment theory states that the way an individual is fostered during infancy and childhood has a profound impact on the way individuals feel and behave when facing stressful situations. According to Bowlby (1973, 1980), proximity-seeking behaviors constitute the primary attachment strategy and represent not only a mean to preserve the physical integrity in threatening situations, but also an inborn affect-regulation device (Cassidy, 1994; Mikulincer, Shaver, & Pereg, 2003). When attachment figures are sensible and responsive, inborn inner states are adequately regulated and positive expectations and beliefs about the caregiver’s accessibility as a source of protection develop. When, on the contrary, parents are unavailable or unresponsive to baby's needs, proximity seeking fails to relieve distress. As a result, negative representations of self and others are formed. The set of patterns of expectations and beliefs commonly referred to as attachment styles (Bowlby, 1973; Simpson, Rholes, Oriña, & Grich, 2002) are commonly represented as regions in a two-dimensional space (Brennan, Clark, & Shaver, 1998; Fraley & Waller, 1998; Griffin & Bartholomew, 1994). The first dimension, labeled Anxiety, represents the degree “to which individuals worry about being rejected, abandoned, or unloved by significant others” (Collins & Feeney, 2004, p. 364). The second, labeled Avoidance, “assesses the degree to which individuals limit intimacy and interdependence with others” (Collins & Feeney, 2004, p. 364). Recent studies gave support to the notion that life experiences, other than those related to parenting, might be formative for adult attachment and that styles and orientations, once formed, hardly ever change (Fraley & Roisman, 2019). However, the notion that the attachment system is active over the entire life span and continues to manifest in thoughts and behaviors related to support seeking, is shared by the majority of researchers in the field. Anxiety and Avoidance are associated with specific alternative ways of interpersonal behavior, affect regulation and information processing other than proximity seeking, called secondary attachment strategies. Individuals high in Anxiety are characterized by the use of the so called hyper-activating strategies and display an exaggeration of distress signals with insistent attempts to attain proximity, support and love. Individuals high in Avoidance employ deactivating strategies, whose primary goal is to keep the attachment system deactivated so as to avoid frustration and further distress caused by caregivers unavailability (Cassidy & Kobak, 1988; Mikulincer, Shaver, & Pereg, 2003).

Research on adult attachment has developed two distinct methodological cultures, characterized by specific research instruments and scientific interests. The "developmental" culture, which relies on the use of the Adult Attachment Interview (AAI), is interested in describing the individual's state of mind regarding childhood experiences with caregivers (Hesse, 2008; Roisman et al., 2007). The socio-psychological culture, which relies on self-report instruments, studies mainly attachment-related thoughts and feelings in adult relationships (Cassidy & Shaver, 2002; Simpson & Rholes,1998). The last approach, to which the present study was inspired, is guided by an explaining model in which attachment dimensions are assumed to influence attachment-related behaviour primarily under conditions of stress or threat (Fraley & Shaver, 1997; Kim, 2006; Maunder, Lancee, Nolan, Hunter, & Tannenbaum, 2006; Mikulincer, 1998; Mikulincer, Birnbaum, Woddis, & Nachmias, 2000).

The relationships between self-report measures of adult attachment dimensions and subjective as well as the physiological response to threatening or attachment related stimuli have been extensively investigated in well-controlled experimental settings. Most of these studies used presentation of short-lived stimuli (Carpenter & Kirkpatrick, 1996; Dewitte, 2011; Mikulincer et al., 2000; Pereg & Mikulincer, 2004), standard stressor tasks or discussions with romantic partners about topics related to their relationship. An interview about painful childhood experiences may represent a more effective instrument to study reactions to attachment related stressor. The experience of being interviewed about personal topics may be in itself a challenging experience, especially for insecure individuals, and, for this reason, affecting their ability to activate mental representations of attachment figures (Mikulincer, Gillath, & Shaver, 2002). Second, unlike in couple interviews, the unavailability of an attachment figure makes the co-regulation processes impracticable, forcing the interviewed to rely on self-regulation abilities. Third, as attachment orientations develop from early experiences with caregivers, one would reasonably expect that the recall of painful childhood experiences to trigger more effectively the regulation strategies. Diamond, Hicks, and Otter-Henderson (2006), among other stimuli, used a brief interview in which participants were requested to provide descriptive adjectives of their parents. Scholars from developmental tradition, used the AAI with the aim to explore associations between attachment strategies assessed through this interview and autonomic reactivity in individuals or couples (Dozier & Kobak, 1992; Holland & Roisman, 2010; Roisman, 2007; Roisman, Tsai, & Chiang, 2004).

However, recent findings showed that developmental and socio-psychological measures predict distinct aspects of adult functioning (Roisman et al., 2007). Unlike self-report attachment instruments, measures from developmental research tradition were not found associated with interpersonal behaviour under conditions of attachment-related threat (Fraley & Shaver, 1998; Simpson et al., 1996).

Since avoidant individuals have more likely experienced episodes of neglect and lack of parental care during childhood, the recollection of painful experiences triggered by an interview should activate an attempt to suppress feelings and thoughts evoked by these memories. When emotional and cognitive load becomes too high, defense lines based on deactivation and suppression should fail; the suppressed contents should break into consciousness leading to the activation of the attachment system and the re-emergence of associated negative expectations about caregiver accessibility (Diamond, Hicks, & Otter-Henderson, 2006). The conflict between these contrasting instances, in turn, should cause sympathetic system activation indexed by skin conductance level (SCL) increase. Under the same conditions, individuals high in Anxiety should not show this response pattern. They, indeed, are less at risk of experiencing conflicts between the drive to approach and expectations of rejection, because they share less negative beliefs about caregiver accessibility as a source of protection and support (Bowlby, 1973; Simpson et al., 2002).

Given that childhood memories have negative valence for individuals with insecure attachment, both Anxiety and Avoidance should be associated with heart rate (HR) increase as a sign that the individual is approaching intense and potentially painful stimuli (Andreassi, 2000; Hare, 1973). Since in everyday situations they tend to avoid the recollection of painful childhood memories, a more pronounced increase is likely to occur among avoidant individuals when they are “forced” by an interview focused on these kinds of critical experiences. Several evidences exist suggesting that individuals high in Avoidance show SCL increase as a sign of emotional conflict when interviewed about attachment related issues (Dozier & Kobak, 1992). Results regarding the role of attachment dimensions effects on cardiac reactivity are contrasting (Kim, 2006; Feeney & Kirkpatrick, 1996; Mikulincer, 1998; Roisman, 2007). However, individual’s reactions to an interview may depend on aspects unrelated to its specific content. Also, stimuli novelty, cognitive effort required to respond to the questions, use of pattern of disengaging attention from emotionally relevant stimuli, or suppressing negative thoughts and feelings may affect emotion response (Brosschot & Janssen, 1998; Dewitte, 2011; Gillath, Bunge, Shaver, Wendelken, & Mikulincer, 2005; Gross & Levenson, 1993).

Because the magnitude of responses depends not only on the salience of topics, but also on the ability to regulate evoked emotions (Balzarotti, Biassoni, Colombo, & Ciceri, 2017; Diamond & Hicks, 2005), we expected that a higher baseline vagal tone would weaken the associations of Avoidance with increase in SCL and HR during the recall of potentially painful childhood memories.

A considerable amount of evidence supports the hypothesis that Avoidance is associated with a decrease, or lack of increase, of self-reported negative emotions evoked by attachment-related content (Diamond et al., 2006). Individuals with high Anxiety scores, instead, have shown heightened subjective emotional response and proneness to experience negative emotions (Diamond & Hicks, 2005; Pereg & Mikulincer, 2004). Thus, anxious individuals should report higher anger, anxiety, and depression after interviews on attachment related-topics. Recent findings have suggested that vagal tone can affect not only the physiological component of emotions, but also the ability to regulate self-reported emotions (Geisler, Kubiak, Siewert, & Weber, 2013; Miller, Kahle, & Hastings, 2017; Scott & Weems, 2014). Thus, we expect that consideration of this confounding factor will allow associations between attachment dimensions and subjective ratings to emerge more clearly.

Among the studies that used interviews as stimuli, few have provided for control task, or controlled for the order in which they were presented. In an attempt to overcome these limitations, I adopted a crossover design that included the administration of two interviews of different content in a counterbalanced order. The interviews comprised of questions that evoked emotionally relevant stimuli and were conceived to intensively engage emotion regulation processes. However, only one of the two interviews required the recall of potentially painful childhood memories.

## Method

### Participants

Women have more intense reactions than males to stimuli evoking negative emotions, and most notably to the social stimuli (Diamond et al., 2006; Kim, 2006; Kring & Gordon, 1998; Whittle, Yucel, Yap, & Allen, 2011). For this reason, this study explored the associations between adult attachment dimensions and emotional response patterns by focusing on the female perspective. The sample was recruited through information sheets or direct contact with individuals enrolled in mandatory courses for a degree in Psychology at the University of Cagliari. Individuals who expressed interest in participating were informed that the aim of the research was to ‘assess relations between self-report and physiological measures of emotions’. Next, the participants completed a brief interview to collect biographical data and information on medical history. Participants who reported taking medication that affected autonomic nervous system (ANS) and central nervous system (CNS), were smokers (Roy, Steptoe, & Kirschbaum, 1994), or suffered from cardiovascular, neurological, endocrine, or mental disorders were excluded (*n* = 31). Eight participants were excluded at a later stage because of data loss due to software or hardware failure during the recording of physiological parameters. The final sample was *N* = 100 women, aged between 21 and 34 years (*M* = 26.22, *SD* = 2.90). Of these women, 43 and 57 possessed a high school diploma and a master's degree, respectively.

### Procedure

Participants approved their participation by signing an informed consent form that described in detail the phases of the experiment and measures collected. The researcher informed the participants that they had the right to end participation in the research at any time and instructed them to refrain from consuming caffeinated beverages or alcohol within 2 hours before the experiment. The experimental procedure began with the administration of questionnaires that assessed adult attachment orientation and level of psychological symptoms over the past week. Before acquiring baseline physiological parameters, a few minutes were dedicated to verify the quality of bio-signal acquisition and allow participants to become familiar with the experimental setup. Next, the participants responded to two interviews of approximately 12 minutes, followed by rest phases of approximately 5 minutes. The order of the interviews was counterbalanced between subjects, but the assignation was not random. Half of the sample were assigned to the AB (interview about movies [FLM] - interview addressing attachment related topics [ATT]) sequence, and the other half of the sample were assigned to the BA sequence (ATT – FLM). During baseline and rest phases, participants assessed the level of subjective emotions alone in the room. Cardiac and electro-dermal signals were continuously monitored. Respondents had no feedback on their physiological activity. At the end of the experiment, the interviewer conducted debriefing interviews in which the interviewees had an opportunity to obtain more information on the purpose of the research, and to discuss their reaction to the experimental procedure. Before discharge, participants were requested to not disclose details to anyone about the procedure. The study was compliant with the Code of Ethics of the Psychological Association of the State in which it was conducted. Although the project was not a part of a funded research programme, the scientific committee of the Association of Psychotherapy of which the author is a member approved the study.

### Content of the Interviews

To ensure that interviews elicited emotion responses of adequate intensity, both were conceived as tasks that requested participants to respond, for several minutes, to a list of questions and demands at a pressing pace. Questions contained in the ATT were similar to those included in the AAI (Hesse, 2008) and addressed experiences potentially occurring in the period from early childhood to adolescence. Because the aim of the study was to assess specific relations between attachment secondary strategies and emotional responses to attachment-related threats, questions focused on participants’ reactions during experiences of separation, threat, loss, rejection, abuse, or neglect (Table 1). When responding to the questions, participants were requested to recount specific episodes that actually occurred to give concrete examples of the reactions and behaviours recalled. In the FLM, participants were asked to choose their top five movies among those they had liked more, indicate the reasons for choice, and sort the movies in order of preference. Because FLM would have probably activated more pleasant experiences than the ATT interview, the first was proposed as a cognitive task of moderate difficulty to ensure that both evoked a comparable arousal level. The interviewer addressed questions or exposed instructions using exactly the same phrases in predetermined order. The interviewer never asked for clarifications and did not comment on the participant’s experiences and choices. In case of request for further information, the interviewer provided only instructions strictly necessary to complete the tasks.

**Table 1:** Questions of the Interview Addressing Attachment Related Topics

I’m going to be interviewing you about your experiences with parents. We'll focus mainly on the period from your earliest childhood memories until adolescence. Please, when responding to the questions, describe specific episodes that actually happened to provide concrete examples of your reactions to the circumstances recalled.	
No.	Question
1.	How did you behave when you were upset as a child? For example, what happened if you were sick?
2.	How did you behave when you were hurt physically, for example an incident when you were playing?
3.	How did you behave when you were upset emotionally?
4.	How did you behave when you needed to be comforted?
5.	Did your parents hold you in their arms when you were upset, or hurt, or ill?
6.	How did you react the first time you were separated from your parents?
7.	When you were little, did you ever feel rejected, pushed away, or ignored by your parents, even if they didn't have the intention or didn't realize it?
8.	Recall an episode when your parents got very angry with you
9.	Recall an episode when you got very angry with your parents
10.	Did your parents ever threaten you, for discipline, or even jokingly? For example, have they ever threatened to abandon you, or to use methods of punishments that made you feel rejected, hurt or ashamed?
11.	Did you ever feel scared, unsafe, or not adequately protected in your family?
12.	When you were young, did you experience the loss of a loved one? How did you react?

### Psychological Measures

The Italian version of the Experiences in Close Relationships (ECR) questionnaire comprises 36 items and assesses individual differences in attachment-related Anxiety and Avoidance (Brennan, Clark, & Shaver, 1998; Picardi et al., 2002): Cronbach’s alpha coefficients were, respectively, .85 and .88 (Bartholomew & Horowitz, 1991; Griffin & Bartholomew, 1994).

The Symptom Checklist-90 (SCL-90; Derogatis, 1977), is a self-report instrument for assessment of general psychopathology and comprises 90 items rated on a scale from 0 (*not at all*) to 4 (*very much*), and 9 clinical scales. In this study, we used the Global Symptom Index (GSI), as the overall distress indicator (Cronbach’s alpha coefficient = .98).

The Profiles of the Mood States (POMS) is a tool that measures the effects of the experimental conditions on emotions in healthy subjects (McNair, Lorr, & Droppleman, 1981). The Italian version comprises 58 adjectives or short phrases that the subject rates on a five-point Likert-type scale. Only the tension-anxiety (t), anger-aggression (a), and depression (d) scales were used. The Cronbach’s alpha coefficients were, respectively, .80, .86, and .81.

### Physiological Measures

An acquisition device equipped with two channels (H&E, Elemaya, Milan), performed the amplification, filtering, digitization, signal processing and data storage of physiological response. A dedicated software running on a personal IBM PC allowed visual inspection for artifacts detection. To measure SCL two Ag/AgCl electrodes (20 × 15 mm) were fastened to the second phalanx of the fourth finger and forefinger of the dominant hand (Andreassi, 2000; Boucsein, 1992; Dawson, Schell, & Filion, 2007). A photoplethysmographic sensor was placed on the tip of the third finger of the same hand. The cardiac and electro-dermal signals were continuously recorded at 35 Hz and 10 Hz, respectively, during baseline, interviews, and rest phases.

In psychophysiological research, abilities to regulate emotions are generally operationalised with heart rate variability (HRV) measures. Two measures were determined from the power spectral analysis of heart rate variability acquired during the baseline and rest phases: High frequency (HF) and low frequency (LF). These measures quantify the frequencies between 0.04 - 0.15 Hz and 0.15 - 0.4 Hz, respectively (Porges, Doussard-Roosevelt, & Maiti, 1994; Singh, Vinod, & Saxena, 2004). Frequency bands were calculated in milliseconds squared and converted to a logarithmic scale. LF is an index of sympathetic activity whereas HF reflects the parasympathetic vagal influences on heart activity (Appelhans & Luecken, 2006; Porges, 1991, 2009). Higher vagal tone levels are hypothesised to co-vary with self-regulatory capacity, good autonomic balance and flexibility to manage environmental challenges and physiological demands (Balzarotti et al., 2017; Friedman & Thayer, 1998; Park & Thayer, 2014). A vagal tone increase during task demands is associated with self-regulatory effort (Butler, Wilhelm, & Gross, 2006; Geisler et al., 2013; Thayer & Lane, 2009). Studies have found contrasting associations between attachment orientations and autonomic regulation and balance (Diamond & Hicks, 2005; Maunder et al., 2006). Two new measures based on the HF values were computed: The between-person (HF_BP_) and within-person (HF_WP_) part of the HF person-mean-centering effects, (Hoffman, 2015, p. 338). HF_BP_ was computed as HF_imean_ - HF_iavg_, where HF_iavg_ was a centering constant based on the HF_imean_ observed distribution. HF_WP_ was computed as HF_ti_–HF_imean_, where HF_ti_ represents the time-invariant HF value at time t for person i. HF_imean_ is the person mean of time-varying HF across the three phases of the experiment. Positive values for HF_WP_ indicated a higher-than-usual HF level at time t for person i.

### Data Analysis

Results regarding effects of attachment dimensions, content, and sequence of the interviews on physiological and subjective responses were analysed using a hierarchical model. Each participant's repeated measures (Phase) were nested within the higher level of analysis (Individuals), which, in turn, was nested within the Sequence level (AB, BA). For calculations regarding SCL and HR, dependent variables were the participant responses over the five phases of the experiment. Data were analysed using two separate generalized linear mixed models (GLMM), because this procedure allows the handling of correlated data and with unequal variances, and overcomes problems related to possible violations of the normality assumption. To take into account the individual differences in intercepts, Individuals nested within Sequence were entered as random factors. The fixed effects part of the model included the categorical main effects: Phase (baseline, FLM, FLM-rest, ATT, ATT-rest) and Sequence (AB, BA); the covariates: Anxiety, Avoidance, baseline HF; and the interactions: Phase × Sequence, Avoidance × Phase, Anxiety × Phase, Avoidance × Anxiety. All the covariates were mean centred around their sample mean (Delaney & Maxwell, 1981). The baseline was considered the centring point of the Phase predictor. Post-hoc analyses were performed using the Sidak adjustment for pairwise comparisons. Maximum restricted likelihood (REML) was used as an estimation method. The contribution of random effects inclusion was tested using the Bayesian information criterion (BIC). Two separate measures of goodness-of-fit (*R*^2^) were computed: One for the conditional model and another one for the fixed part of the model (Gelman & Hill, 2006, p. 474).

Models used to test the effects on LF, HF and subjective responses were the same as for SCL and HR, except that the repeated measures were three (Baseline, FLM-rest, ATT-rest) and baseline HF was not included among predictors. HF_BP_ and HF_WP_ were added to the list of predictors of subjective responses.

Normality assumption was verified, for each phase, by residuals histogram inspection and Kolmogorov-Smirnov test use. For diagnostic purposes, calculations were repeated after replacement of outliers with each phase mean value. All the calculations were performed using SPSS (Version 21.0) for Windows statistical package. Analyses regarding SCL were repeated using the option available in the GENLINMIXED procedure designed to obtain robust fixed effects estimations.

## Results

### General Statistics

Table 2 reports general statistics of the sample by sequence assignation. According to the classification of Bartholomew and Horowitz (1991), and Griffin and Bartholomew (1994), 67 participants were secure regarding adult attachment orientation, 20 were dismissing, 10 preoccupied, and 3 fearful. Only 16 participants reported SCL-90 scores higher than the cut-off point for caseness identification (.90). As Table 3 shows, at the start of the experiment, participants reported low to medium baseline depression, anxiety, and rage T scores.

**Table 2:** Characteristics of the Participants Assigned to the Normal and Inverted Sequence of the Interviews (N = 100)

Variable/Value	Sequence							
	AB (*n* = 50)				BA (*n* = 50)			
	*M*	*SD*	*N*	%	*M*	*SD*	*N*	%
Age	25.58	2.35			26.90	3.07		
ECR Avoidance	45.88	14.65			48.66	20.42		
ECR Anxiety	66.22	17.08			67.58	16.82		
SCL-90 Total Score	44.62	28.82			47.32	38.73		
SCL-90 GSI caseness^a^			8	14			9	18
Marital status								
Married/engaged			31	62			28	56
Single			13	26			18	36
N.R.			6	12			4	8
Education								
Graduate			25	50			18	36
Master's Degree			25	50			32.	64
Attachment style^b^								
Secure			35	70			32	64
Dismissing			8	16			12	24
Preoccupied			5	10			5	10
Fearful			2	4			1	2

*Note*. A = interview about movies; B = interview addressing attachment related topics; ECR = experiences in close relationships; SCL- 90 = symptom checklist-90; GSI = global severity index; N.R. = missing or not responding.

^a^Classification based on SCL-90 Global Severity Index score > .90. ^b^Classification based on mean +1 *SD* of Anxiety and Avoidance scales of the Experiences in Close Relationships.

**Table 3:** Physiological and Subjective Measures of Emotion During the Experiment (N = 100)

Sequence/Measure	Baseline		FLM		Rest		ATT		Rest	
	*M*	*SD*	*M*	*SD*	*M*	*SD*	*M*	*SD*	*M*	*SD*
AB (*n* = 50)										
SCL	5.76	2.54	9.98	3.83	6.81	2.73	9.52	4.35	6.51	2.41
HR	84.00	11.01	86.50	10.02	81.50	9.63	84.80	8.30	80.55	9.21
POMS-d	48.90	6.48			46.94	5.44			47.18	5.66
POMS-t	49.00	9.82			47.04	10.05			44.96	11.46
POMS-a	46.22	8.53			44.00	5.91			45.06	8.10
LF	6.64	0.91			7.02	0.89			7.17	0.89
HF	6.08	1.01			6.40	0.96			6.53	0.87
HF_BP_	1.71	0.12			1.71	0.12			1.71	0.12
HF_WP_	-0.25	0.06			0.06	0.06			0.19	0.06
BA (*n* = 50)										
SCL	5.18	2.53	8.62	4.51	6.81	4.48	9.07	4.44	6.40	3.23
HR	86.00	12.29	84.17	9.32	81.30	29.43	87.18	10.79	82.22	10.26
POMS-d	47.46	6.64			46.22	6.08			47.90	6.07
POMS-t	44.38	8.16			41.42	11.88			44.24	14.14
POMS-a	42.84	4.36			42.20	3.77			43.30	5.22
LF	6.35	0.96			7.08	0.87			7.08	0.87
HF	7.72	1.04			6.44	0.89			6.42	0.90
HF_BP_	1.56	0.12			1.56	0.12			1.56	0.12
HF_WP_	-0.47	0.06			0.24	0.06			0.22	0.06

*Note.* FLM = interview about movies; ATT = interview addressing attachment related topics; AB = FLM-ATT sequence; BA = ATT-FLM sequence; SCL = skin conductance level; HR = heart rate; POMS-d, POMS-t, POMS-a = depression, tension, and anger T scores of the profile of mood states; HF_BP_ = between-person part of the HF person-mean-centring effects; HF_WP_ = within-person part of the HF person-mean-centring effects.

Participants assigned to the AB sequence were younger than those assigned to the BA, mean difference = -1.32, *SE* = 0.55, *p* = 0.17; had higher POMS-t, mean difference = 2.52, *SE* = 0.93, *p* = .008, and POMS-r baseline scores, mean difference = 2.48, *SE* = 1.0, *p* = .015. SCL was higher during the interviews than the following rest phases of 2.66 μS, *SE* = 2.41, *p* < .001. HR increased by 4.27 bpm, *SE* = 0.58, *p* < .001. There was no inequality in the carryover effect on the HR and SCL responses by sequence.

### Adult Attachment Effects on Physiological Response

**Figure 1:**
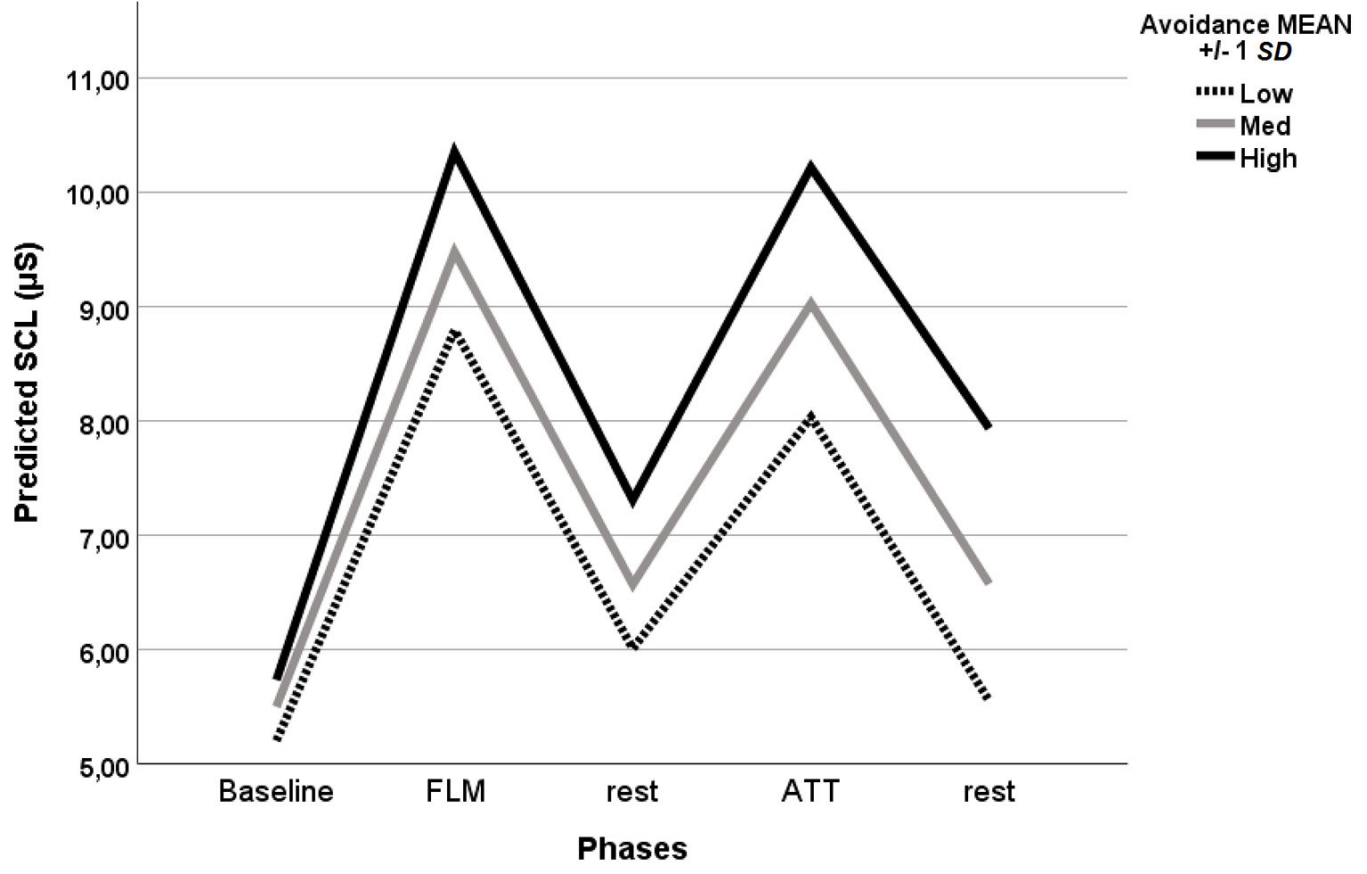
Mean predicted skin conductance level during the experiment in participants with low, medium and high Avoidance level (±1 *SD*) *Note.* SCL = skin conductance level; FLM = interview about movies; ATT = interview addressing attachment related topics.

The best-fit model included a diagonal matrix structure for repeated measures and a random intercept. Baseline SCL was lower than during the following phases of the experiment. Both interviews evoked comparable levels of arousal, higher than those observed in rest phases regardless of sequence. Table 4 summarizes the GLMM results regarding fixed effects. During FLM, SCL increased from the baseline by 3.84 μS, *SE* = 0.39, *p* < .001, whereas during ATT an increase of 3.37 μS was observed, *SE* = 0.43, *p* < .001. Although there was an Avoidance main effect, *p* = .024, a significant Avoidance × Phase interaction indicated that Avoidance was associated with a SCL increase during the ATT interview of 0.04 μS, *SE* = 0.02, *p* = .003, and during ATT-rest phase of 0.05 μS, *SE* = 0.02, *p* = .003. There was also a marginally significant Avoidance × Anxiety effect, *p* = .08. A further GLMM using a robust estimate of fixed effects was performed controlling for the participants’ age and removing predictors with non significant effects (Anxiety, Anxiety × Phase, and Avoidance × Anxiety effect). Results revealed significant Phase, *F*(4, 483) = 58.65, *p* < .001, Avoidance, *F*(1, 483) = 8.491, *p* = .004, Avoidance × Phase, *F*(4, 483) = 2.54, *p* = .039 and Age effects, *F*(1, 483) = 4.69, *p* = .031. Avoidance was associated with SCL increases of 0.041 μS during ATT, *SE* = 0.015, *p* = .006; 0.049 μS during ATT-rest, *SE* = 0.026, *p* = .061; 0.026 μS during FLM, *SE* = 0.014, *p* = .059, and 0.021 μS, during FLM-rest phase, *SE* = 0.011, *p* = .057. Age and HF were associated with lower SCL, -0.25, *SE* = 0.11, *p* = .027 and -0.47, *SE* = 0.26, *p* = .072, respectively. Corrected model *F*(16, 483) = 19.79, *p* < .001 (See Figure 1).

**Table 4:** Effects of Adult Attachment Dimensions and Content of the Interviews on Physiological Measures of Emotion During the Experiment (Generalized Linear Mixed Model Fixed Effects)

Measure/Factor and Covariate	*F*	*df*1	*df*2	*p*	-2 LL	BIC	*R*^2tot^	*R*^2fix^
SCL								
Corrected model	17.05	21	478	< .001	2314.48	2351.49	.76	.26
Sequence	2.09	1	478	.149				
Phase	81.66	4	478	< .001				
Sequence × Phase	1.39	4	478	.238				
Baseline HF	2.30	1	478	.131				
Avoidance^a^	5.10	1	478	.024				
Anxiety^a^	0.31	1	478	.577				
Phase × Avoidance^a^	2.57	4	478	.037				
Phase × Anxiety^a^	0.65	4	478	.629				
Anxiety^a^ × Avoidance^a^	3.08	1	478	.080				
HR								
Corrected Model	8.03	21	478	< .001	3197.10	3234.12	.85	.27
Sequence	0.35	1	478	.555				
Phase	29.45	4	478	< .001				
Sequence × Phase	4.11	4	478	.003				
Baseline HF	29.96	1	478	< .001				
Avoidance^a^	0.70	1	478	.402				
Anxiety^a^	0.26	1	478	.614				
Phase × Avoidance^a^	0.52	4	478	.724				
Phase × Anxiety^a^	0.18	4	478	.949				
Anxiety^a^ × Avoidance^a^	0.03	1	478	.865				
HF								
Corrected Model	6.50	12	287	< .001	727.29	735.43	.81	.08
Sequence	1.16	1	287	.283				
Phase	30.79	2	287	< .001				
Sequence × Phase	3.00	2	287	.052				
Avoidance^a^	0.04	1	287	.844				
Anxiety^a^	0.19	1	287	.666				
Phase × Avoidance^a^	0.04	2	287	.965				
Phase × Anxiety^a^	2.85	2	287	.060				
Anxiety^a^ × Avoidance^a^	3.11	1	287	.079				
LF								
Corrected Model	6.22	12	287	< .001	729.95	752.48	.78	.10
Sequence	1.06	1	287	.304				
Phase	31.26	2	287	< .001				
Sequence × Phase	3.03	2	287	.050				
Avoidance^a^	0.41	1	287	.521				
Anxiety^a^	0.20	1	287	.657				
Phase × Avoidance^a^	0.15	2	287	.864				
Phase × Anxiety^a^	1.54	2	287	.216				
Anxiety × Avoidance^a^	1.43	1	287	.233				

*Note.* SCL = skin conductance level; HR = heart rate; HF = high frequency of heart rate variability; LF = low frequency of heart rate variability; BIC = Bayesian information criterion; *R*^2tot^ = *R*^2^ for conditional model; *R*^2fix^ = *R*^2^ for the fixed part only; -2LL = -2 Log Likelihood.

^a^Avoidance and Anxiety scales of the experiences in close relationships.

The best-fit model for HR had a diagonal covariance matrix structure for repeated measures and a random intercept. HR during FLM-rest phase was lower of -4.71 beat per minute (bpm) than during baseline, *SE* = 0.86, *p* < .001; during ATT-rest phase was lower of -3.8 bpm, *SE* = 0.88, *p* < .001. Baseline resting vagal tone was associated with a HR mean decrease of -4.67 bpm, *SE* = 0.82, *p* < .001. When calculations were performed using a robust effects estimate, direction of effects and their statistical significance did not change. Both LF and HF significantly increased from baseline. During FLM-rest phase, the HF mean increase of 0.51, *SE* = 0.08, *p* < .001, during FLM-rest phase the increase was 0.57, *SE* = 0.08, *p* < .001. There was also a Sequence × Phase effect, but pairwise contrasts were not significant. The LF mean increase during the ATT-rest phase was = 0.37, *SE* = 0.11, *p* < .001, during the ATT-rest phase the increase was 0.53, *SE* = 0.11, *p* < .001. A Sequence × Phase effect emerged but coefficients estimates were non significant. Analyses repeated with robust estimates yielded similar results.

### Attachment Dimensions Effects on Subjective Measures

Table 5 summarizes the GLMM estimates of fixed effects on subjective ratings of anger, depression and tension experienced during baseline and rest phases of the experiment. Attachment dimensions, between-person (HF_BP_), and within-person (HF_WP_) time-varying HF did not affect subjective measures of emotions. There were Sequence and Sequence × Phase significant effects on the POMS anger scores. Participants assigned to the AB sequence reported higher scores, mean difference = 1.91, *SE* = 0.80, *p* = .017. Anger was lower after the FLM interview than baseline, with mean difference in the AB sequence = -1.01, *SE* = 0.36, *p* = .009. There was a significant effect of Phase on POMS depression scores. Baseline depression scores were higher than after the FLM interview, mean difference = -1.68, *SE* = 0.56, *p* = .003, and ATT interviews, mean difference -1.39, *SE* = 0.73, *p* = .057. There were Sequence and Sequence x Phase effects on POMS tension-anxiety scores. Participants assigned to the AB sequence reported lower anxiety in the FLM-rest phase compared with baseline, mean difference = -1.68, *SE* = 0.73, *p* = .043. Analyses repeated with robust estimates yielded similar results.

**Table 5:** Effects of Adult Attachment Dimensions and Content of the Interviews on Subjective Rating of Anger, Depression, and Tension During the Experiment (Generalized Linear Mixed Model Fixed Effects)

Measure/Factor and Covariate	*F*	*df*1	*df*2	*p*	-2 LL	BIC	*R*^2tot^	*R*^2fix^
POMS-a								
Corrected Model	2.50	14	285	.002	1650.99	1673.60	.69	.12
Sequence	5.73	1	285	.017				
Phase	5.85	2	285	.003				
Sequence × Phase	1.96	2	285	.143				
Avoidance^a^	0.36	1	285	.549				
Anxiety^a^	0.97	1	285	.325				
Phase × Avoidance^a^	0.036	2	285	.965				
Phase × Anxiety^a^	1.80	2	285	.167				
Anxiety × Avoidance^a^	2.56	1	285	.111				
HF_BP_	0.39	1	285	.535				
HF_WP_	0.19	1	285	.663				
POMS-d								
Corrected Model	2.28	14	285	.006	1739.27	1761.88	.76	.10
Sequence	0.41	1	285	.521				
Phase	5.53	2	285	.004				
Sequence × Phase	1.96	2	285	.143				
Avoidance^a^	0.37	1	285	.543				
Anxiety^a^	1.98	1	285	.160				
Phase × Avoidance^a^	0.66	2	285	.515				
Phase × Anxiety^a^	0.49	2	285	.611				
Anxiety^a^ × Avoidance^a^	2.15	1	285	.143				
HF_BP_	91.06	1	285	.341				
HF_WP_	0.24	1	285	.622				
POMS-t								
Corrected Model	2.102	14	285	.012	1769.11	1791.25	.73	.10
Sequence	4.27	1	285	.040				
Phase	1.87	2	285	.155				
Sequence × Phase	4.75	2	285	.009				
Avoidance	2.41	1	285	.122				
Anxiety	2.03	1	285	.155				
Phase × Avoidance	1.29	2	285	.278				
Phase × Anxiety	0.37	2	285	.688				
Anxiety × Avoidance	0.07	1	285	.796				
HF_BP_	0.44	1	285	.508				
HF_WP_	0.01	1	285	.923				

*Note*. POMS-d, POMS-t, POMS-a = depression, tension and anger T scores of the profile of mood states; HF = high frequency of heart rate variability; *R*^2tot^ = *R*^2^ for conditional model; *R*^2fix^ = *R*^2^ for fixed part only; BIC = Bayesian information criterion; HF_BP_ and HF_WP_ = between and within-person parts of the person-mean-centring effects of time-varying HF; -2LL = -2 Log Likelihood.

^a^Avoidance and Anxiety scales of the experiences in close relationships.

## Discussion

The first hypothesis was that Avoidance would have been associated with an SCL increase during the recall of childhood memories. According to the hypotheses, only Avoidance was associated with SCL increase during the ATT, but not during the FLM interview, whereas baseline vagal tone did not mediate or moderate participants’ responses. Unexpectedly, SCL increased during both the ATT interview and ATT-rest phase, suggesting that Avoidance enhanced reactivity and impaired the ability to recover. However, a close examination of robust estimates of effects, revealed that Avoidance effects on SCL did not differ much through the experiment. This finding raises the problem of the ability of the experimental conditions to elicit attachment specific responses. There are several explanations for why electrodermal response of avoidant participants during the two interviews differed to a lower extent than expected. First, the ATT interview may have not sufficiently activated the attachment system. Although the interview focused on memories of problematic experiences, the interviewer did not make any attempt to actively direct the participant’s attention on the topics. Participants could have omitted painful memories or used narratives that may have helped them to trivialise or minimise relevance of the experienced episodes. Second, we cannot rule out that the FLM interview also activated the attachment system, even if to a lesser extent, reducing differences in responses. Regarding the nature of the association between Avoidance and participants’ electrodermal response, the SCL increase may be have been a mere physiological correlate of external threats perceived in the experimental procedure, such as imaginings about the interviewer’s opinion, or a reflex of the strive to suppress external manifestations of emotions evoked by the interviews. In summary, many other factors than attachment secondary strategies, difficult to disentangle, have likely been simultaneously activated by both experimental conditions. Overall, the results partially agree with those reported by studies that have used attachment-related stimuli (Diamond et al., 2006) and suggest that the more avoidant individuals reacted to the recall of memories of personal issues with a pattern of generalised increase of physiological arousal and a reduced recovery.

Contrary to expectations, no significant adult attachment dimension effect on cardiac response emerged, alone or interacting with the content of the interviews. Differences observed in SCL and HR and response profiles, may have depended on different mechanisms underlying the activation and regulation of these physiological parameters. The SCL is a measure of pure sympathetic activity, whose regulation, mediated by the endocrine system, allows relatively slow adjustments (Dawson, Schell, & Filion, 2007). Although both branches of the ANS affect heart activity, HR regulation is mediated mainly by the parasympathetic branch of the ANS, more specifically via the vagus nerve; a means that allows faster and effective adjustments. Therefore, participants’ ability to regulate arousal may have masked the attachment dimensions’ effects on HR reactivity. Accordingly, baseline vagal tone provided the most relevant contribution to HR (Singh et al., 2004), suggesting that it should be regarded as a critical factor to be controlled in experiments using HR as a dependent measure. However, unlike what Diamond and Hicks (2005), and Maunder and colleagues (2006) observed, attachment dimensions did not significantly affect HRV measures, and vagal tone did not affect relationships between attachment and cardiac response.

The results did not provide evidence of significant effects of attachment dimensions on subjective ratings of emotion during the baseline and rest phases. Diamond et al. (2006) reported the same findings using a similar stress task. Neither the individual’s average level of HF across experimental phases, nor its changes around to the individual’s mean significantly affected subjective ratings. Thus, no data emerged in support to the hypothesis that vagal tone moderates attachment effects on subjective response. POMS T scores ranged overall below the normative value for the Italian population. Baseline depression ratings were higher than in the FLM rest phase, whereas baseline tension and anger scores were higher only in participants assigned to the AB sequence. The results suggest that participants reported to feel relaxed and in good spirits after having familiarized with the experimental procedure. It is worth to note that participants rated their emotions while they were alone in the room; this may have allowed them to distance themselves from the stress evoked by the presence of the interviewer. Furthermore, the act of identifying, verbalizing, and rating inner states may have helped them to reduce the intensity of the arousal. Nevertheless, The LF and HF levels recorded at the same time did not confirm a coherent pattern of recovery, but showed a contemporary increase in sympathetic and parasympathetic activity, signals of the participants' effort sustained to cope with the stress, and of the parallel activation of regulation processes. The POMS scores showed an opposite trend to those of SCL, HF and LF but similar to that of HR, suggesting that subjective ratings and HR were more influenced by the conscious attempts to mask or regulate the arousal.

The results of this study support the assumption that avoidant individuals are at risk of showing an increase of sympathetic activity when interviewed about personal issues, even if the interview is not addressing attachment-related topics, whereas their subjective ratings are indistinguishable from those of the less avoidant individuals. The results agree with recent evidence showing that avoidant individuals defensively limit the processing of potentially distressing information (Edelstein, 2006). At an early stage, when encoding emotional information, they tend to use more cognitive resources, then, at a next stage, employ post-emptive strategies aimed at suppressing the accessibility of previously encoded emotional information (Zheng et al., 2015). Furthermore, the pattern of physiological response observed suggests that avoidant participants likely experienced sensations of discomfort at a somatic level, even if they did not report them because of their inability to identify or verbalise them and the attempt to suppress them. These sensations of discomfort, the low proximity seeking and the automatic avoidance tendency (Dewitte et al., 2008) may contribute to the explanation of why they are less willing to seek psychological help for their problems (Dozier, 1990).

### Limitations and Future Directions

Not performing random assignment to the sequences is a relevant limitation of the study. However, the Sequence effects were weak and limited to self-report measures and to HRV indicators, though non-significant. The hypotheses of the study were based on the assumption that there is a relationship between measures of adult attachment orientations and patterns of response evoked by memories of events far in time, a period in which these orientations are not yet well formed. Perhaps, if the study had explored the associations between states of mind related to childhood memories and physiological responses evoked by these memories, different results would have been observed. The sample composition suggests caution in extending results to male individuals or the general population. Participants were young women with a high level of education and good general health. In addition, the fact that participants were mostly psychology students, may have had relevant effects in shaping participants’ expectations about the experimental procedure.

Because of the technical limitation of the equipment used to acquire cardiac signals, the results based on HRV indicators can have only a limited value. First, signals were acquired through plethysmographic sensors. Second, no information on respiration frequency was collected during the interviews (Schipke, Pelzer, & Arnold, 1999). Third, the sampling frequency for the signal was lower than that suggested by the European Cardiology Task Force to obtain reliable HRV measures (Malik et al., 1996). Relevance of the results should be evaluated in the context of the mind of the study: To obtain useful suggestions for clinical practice. This study focused on the overall level of response as an expression of the individual’s ability to cope with meaningful social interactions. Consistently, the SCL and HR responses were computed as values averaged over the whole interview. Assessing the effects of each question was not of primary interest and would require a more complex and demanding experimental design. In view of the aforementioned limitations, further research could benefit from more precise measures and rigorous experimental designs.
